# Relationship between optical coherence tomography findings and clinical variables in patients with opiate use disorder

**DOI:** 10.5935/0004-2749.20230013

**Published:** 2023

**Authors:** Filiz Özsoy, Müberra Kulu, Yakup Özarslan, Sevda Korkmaz

**Affiliations:** 1 Clinic of Psychiatry, Tokat State Hospital, Tokat, Turkey.; 2 Tokat Mental Health and Diseases Hospital, Tokat, Turkey.; 3 Department of Psychiatry, School of Medicine, Fırat University Elazığ, Turkey.

**Keywords:** Opiates alkaloids, Opioid-related disorders, Tomography, optical coherence, Retinal nerve fiber layer thickness, Macular volume, Alcaloides opiáceos, Transtornos relacionados ao uso de opioides, Tomografia de coerência óptica, Espessura da camada de fibras nervosas da retina, Volume macular

## Abstract

**Purpose:**

This study aimed to examine optical coherence tomography findings in patients with opiate use disorder by comparing them with healthy controls.

**Methods:**

The study included 30 opiate use disorder patients and 30 controls. The participants’ detailed biomicroscopic examinations, visual acuity, intraocular pressure, and both eye examinations were evaluated. A total of 120 eyes were evaluated using optical coherence tomography, measuring the central macular thickness, mean macular thickness, mean macular volume and retinal nerve fiber layer thickness. Moreover, all participants filled in the demographic data form and Barratt Impulsiveness Scale.

**Results:**

Upon examination of the optical coherence tomography findings, central macular thickness, mean macular thickness, and mean macular volume were thinner in both eyes in patients with opiate use disorder (p<0.01 in all measurements in both eyes). Similarly, the total values of the superior quadrant and retinal nerve fiber layer thickness were statistically significant in both eyes compared to that in the control group (p=0.007, p=0.002; p=0.049, p=0.007, in the right and left eyes, respectively). Only the left eye was positively correlated with retinal nerve fiber layer superior quadrant measurement and hospitalization (r=0.380, p=0.039).

**Conclusion:**

Our results revealed that the patients’ central macular thickness, mean macular thickness, and mean macular volume values were thinner. Increase in the retinal nerve fiber layer thickness superior quadrant thickness and total value was also observed. Further studies with larger sampling groups that evaluate neuroimaging findings should be conducted.

## Abstract (pt)

**Objetivo:**

O objetivo foi investigar foi, os achados da tomografia de coerência óptica em pacientes com transtorno do uso de opiáceos, comparando-os com controles saudáveis.

**Métodos:**

O estudo incluiu 30 pacientes com transtorno do uso de opiáceos e 30 controles. Os exames biomicroscópicos detalhados de todos os participantes, acuidade visual, pressão intraocular e ambos os exames oculares foram avaliados com tomografia de coerência óptica. Um total de 120 olhos foram avaliados usando tomografia de coerência óptica, e a espessura macular central, espessura macular média, volume macular médio e a espessura da camada de fibra nervosa da retina dos participantes foram medidos. Além disso, todos os participantes preencheram o Formulário de Dados Demográficos e a Escala de Impulsividade Barratt (BIS-11).

**Resultados:**

Quando os achados de tomografia de coerência óptica foram examinados, espessura macular central, espessura macular média e volume macular médio eram mais finos de acordo com controles saudáveis em ambos os olhos em pacientes com transtorno do uso de opiáceos (p<0,01 em todas as medições em ambos os olhos). Da mesma forma, os valores totais do quadrante superior e espessura da camada de fibra nervosa da retina estavam mais em níveis estatisticamente significativos em ambos os olhos em comparação com o grupo controle (p=0,007, p=0,002; p=0,049, p=0,007, no olho direito e esquerdo, respectivamente). Estar internado em hospital e apenas a medida do quadrante superior da espessura da camada de fibra nervosa da retina do olho esquerdo associou-se positivamente (r=0,380, p=0,039).

**Conclusão:**

Em nossos resultados, descobrimos que os valores de espessura macular central, espessura macular média e volume macular médio dos pacientes eram mais finos. Verificamos também espessamento no quadrante superior e valor total da espessura da camada de fibra nervosa da retina. Nosso estudo deve ser apoiado por novos estudos com grupos de amostragem maiores, nos quais os achados de neuroimagem são avaliados.

## INTRODUCTION

Substance use disorder (SUD) continues to be an important public health issue despite all the precautions taken in our country and worldwide. Substances that cause addiction according to the Diagnostic and Statistical Manual of Mental Disorders (DSM-5) are classified as alcohol, caffeine, cannabis, hallucinogens, inhalants, opiates, sedatives-hypnotics and anxiolytics, stimulants, nicotine, and others (or unknown substances)^(1)^. Opiate, which is used to relieve pain, is one of the oldest known drugs in history. Although opiates were synthesized as legal drugs, it was found that they also had highly addictive effects in the early twenty-first century^(2)^. Globally, the most commonly abused and highly addictive opioid is heroin^(3)^.

Heroin addiction is a chronic neurobiological disease, and it was reported in previous studies that there are genetic, environmental, and familial factors and personality traits in its etiology^(4)^. It was found that people who have opiate use disorders have personality traits like impulsiveness and search for innovation^5)^. In addition, deterioration of executive functions, such as working memory, attention, and planning, is noted^(6)^. There has been significant progress in studies aimed at illuminating the etiology of psychiatric diseases in recent years. Visual pathways have been ideal detecting neurodegenerative processes^(7)^. Consequently, studies have been conducted to follow the changes in retinal nerve networks through optical coherence tomography (OCT) in recent years^(8,9,10,11)^. Central macular thickness (CMT), mean macular thickness (MMT), mean macular volume (MMV) and retinal nerve fiber layer (RNFL) thickness can easily be measured through OCT^(9)^.

Upon review of the literature, it was reported that OCT was used to determine the severity of neurodegeneration in psychiatric disorders like schizophrenia, major depressive disorder, and obsessive-compulsive disorder^(7,9,10)^. Studies conducted to examine OCT findings in patients with alcohol use disorder/SUD are limited ^(11,12,13)^. No studies were found examining OCT findings in patients with opiate use disorder. This study aimed to examine the OCT findings of patients with opiate use disorder compared with that in healthy controls, as well as the relationship between clinical variables such as patients’ age, frequency of hospitalization, legal problems due to opiate use, and impulsiveness.

## METHODS

This study was approved by the Ethics Committee for Non-Interventional Clinical Research of Gaziosmanpaşa University (approval number, 83116987-092; date, 02/21/2020; project number, 20-KAEK-018). The study was conducted in line with the Helsinki Declaration.

### Inclusion and exclusion criteria of participants

A total of 30 patients diagnosed with opiate use disorder according to DSM-5 criteria were treated in Tokat Mental Health and Diseases Hospital Alcohol and Drug Research, Treatment and Training Centre (AMATEM). The inclusion criteria were being literate and volunteering to participate in the study. People with poor general condition, chronic liver disease, chronic renal failure, diabetes, heart disease, another DSM-5 disorder, and other non-opiate SUD/alcohol use disorder and those who did not want to participate in the study were excluded. The healthy control group consisted of patients who matched the study group in terms of demographic data such as age, sex, education status, and who did not have any diagnosed psychiatric disease and alcohol/ substance use at the time of the study or previously. All participants were evaluated by the same ophthalmologist with detailed biomicroscopic examinations in terms of visual acuity, intraocular pressure, and front and rear segment examinations. The inclusion criteria for the participants were having corrected visual acuity logMAR 0.00, intraocular pressure <20 mmHg, and globe axial length 20-24 mm. The exclusion criteria for all individuals who participated in the study were having retinal pathology, cataract, uveitis, corneal disease, ocular trauma history, neurological disorders like optic neuritis, and spherical and cylindrical breakage defects greater than +/-1.00 diopter (dpt).

Detailed eye examination was performed before OCT measurements along with the demographic data form and Barratt Impulsiveness Scale (BIS-11). All participants signed the written informed consent forms.

### Data collection tools

**Sociodemographic deata form:** This is a semistructured form and was prepared by researchers for the purposes of the study. It consists of clinical evaluation questions like age, marital status, education level, working status, previous hospitalization in AMATEM clinic, and legal problems due to opiate use.

**Barratt Impulsiveness Scale (BIS-11):** This scale was developed by Patton et al. and is a 30-point self-notification scale used to evaluate impulsiveness: motor (motor impulsiveness and impatience), attention (carelessness and cognitive irregularity), and planning (cognitive confusion and intolerance). It was reported that as the calculated total score increases, the level of impulsiveness also increases.

**Evaluation of optical coherence tomography (OCT) findings:** OCT measurements were taken by the same ophthalmologist. Both eyes of all participants were assessed. The retinal parameters measured were as follows, CMT, MMT, MMV and RNFL thickness. To analyze macular thicknesses, the scan protocol of the macula (macular cube 512 × 128 protocols) was used. The axial length of both patients and controls were measured using a biometer.

**Statistical analysis:** The SPSS for Windows 22 software (Statistical Package for Social Sciences for Windows 22 SPSS (Inc., Chicago, IL, USA) was used in the calculations. Data distribution was analyzed using the Kolmogorov-Smirnov test. Categorical data were expressed in numbers and percentages, and numerical data as mean ± standard. The chi-square test was used to compare the patients’ categorical data, and independent samples t-test or Mann-Whitney U test was used to compare numerical data. The relationship between the measurements and the scale scores was examined using Pearson correlation (r) analysis. The p values <0.05 was considered statistically significant.

## RESULTS

A total of 50 opiate use disorder patients were interviewed for this study, and 9 people were not included because of their refusal to participate. Among the remaining 41 patients, 4 people were not included in the study because they used other substances along with opiate. One person was excluded due to a psychotic disorder associated with opiate use, 1 person due to chronic liver disease, and 1 person due to diabetes mellitus. Among the remaining 34 patients, 1 patient was excluded due to glaucoma, 2 patients due to hypermetropy, and 1 patient due to cataract.

A total of 60 people were included in our study, and 120 eyes were evaluated. Among these, 30 patients were diagnosed with opiate use disorder according to DSM-5 criteria, and 30 were included in the healthy control group. The eye measurements were conducted on the first day of hospitalization, just before the medical treatment began. The participants who were included in the control group has not previously received psychiatric medical treatment and at the time of the study period. All participants were males. The mean age of the patient group was 26.57 ± 8.35 years, and that of the control group was 28.3 ± 10.64 years (p=0.772). All participants had 1 package/day smoking status. The patient group’s past smoking duration was 6.56 ± 3.05 and 6.33 ± 3.2 in the healthy control (p=0.637). The distribution of participants’ demographic data is given in table 1.Upon evaluating the OCT findings, it was observed that the macula layer thickness, MMT, and MMV were reduced in both eyes compared to that in healthy controls (all p<0.01 in the right and left eyes) (Table 2). Patients with opiate use disorder had statistically and significantly higher values than that in the control group in terms of superior quadrant and total value for RNFL thickness (p=0.007, p=0.002; p=0.049, p=0.007, for the right and left eyes, respectively) (Table 3).

**Table 1:** Sociodemographic characteristics of the participants

	Opiate use disorder patient group (n=30) N (%)	Healthy control group (n=30) N (%)	p value
Marital status			
Single/married	25/5 (83.3/16.7)	24/4 (80/20)	>0.05
Educational status			
Primary school graduate	21 (70)	20 (66.66)	
High school graduate	3 (10)	2 (6.66)	>0.05
University graduate	6 (20)	8 (26.66)	
AMATEM hospitalization			
Yes/no	8/22 (26.7/73.3)	-	
Legal issues due to opiate use			
Yes/no	15/15 (50/50)	-	

The healthy control group did not have any current or past diagnosed psychiatric diseases or diagnosed comorbidities. The chi-square test was used in the calculations.

**Table 2:** Macular layer thickness and volume evaluated using optical coherence tomography

	Opiate use disorder patient group (n=30) (Mean ± SD)	Healthycontrol group (n=30) (Mean ± SD)	p value
Central macular layer thickness			
Right eye	219.5 ± 20.98	249 ± 16.92	<0.01* a
Left eye	214.4 ± 14.18	247.4 ± 19.09	<0.01* a
Mean macular layer thickness			
Right eye	263.9 ± 17.12	286.33 ± 13.7	<0.01* a
Left eye	263.73 ± 14.17	283.17 ± 13.22	<0.01* b
Mean macular layer volume			
Right eye	8.81 ± 0.29	10.28 ± 0.47	<0.01* b
Left eye	8.84 ± 0.31	10.27 ± 0.52	<0.01* b

^a^= Mann - Whitney U test and

^b^= independent sampling *t*-test was used in the calculations.

*p<0.05.

**Table 3:** Retina nerve fiber layer thickness evaluated using optical coherence tomography

Retina nerve fiber layer thickness	Opiate use disorder patient group (n=30) (Mean ± SD)	Healthycontrol group (n=30) (Mean ± SD)	p value
Superior quadrant			
Right eye	128.33 ± 18.97	119 ± 16.98	0.007* b
Left eye	133.27 ± 16.82	115.53 ± 25.89	0.002* a
Inferior quadrant			
Right eye	126.87 ± 15.92	125.77 ± 16.18	0.792^b^
Left eye	132.13 ± 16.57	125.53 ± 16.25	0.125^b^
Temporal quadrant			
Right eye	81.07 ± 12.34	84.43 ± 12.1	0.996^b^
Left eye	72.03 ± 10.08	74.13 ± 12.17	0.885^b^
Nasal quadrant			
Right eye	73.03 ± 14.39	76.83 ± 13.13	0.344^a^
Left eye	75.33 ± 15.93	75.23 ± 13.28	0.976^a^
Total value			
Right eye	101.83 ± 10.48	96.5 ± 10.04	0.049* b
Left eye	103.63 ± 9.7	96.53 ± 9.93	0.007* b

^a^Mann - Whitney U test and

^b^independent sampling *t*-test was used in the calculations.

*p<0.05

When BIS-11 data were evaluated, no statistically significant differences were detected between the groups in terms of motor impulsiveness, impulsiveness in making plans, and the total score of the scale (p values: 0.940, 0.549, 0.072, respectively). However, the subdimension of impulsiveness associated with attention was lower in the patient group (p=0.01). No significant relationships were detected between age, legal issues due to opiate use, and OCT findings in the patient group (p>0.05). In the AMATEM clinic, only the left eye was positively related with RNFL superior quadrant measurement and hospitalization (r=0.380, p=0.039). Upon evaluating the relationship of BIS-11 scores and OCT findings with Pearson correlation analysis, a negative correlation was observed in the right eye total RNFL value, BIS-11 attention-related impulsiveness subdimension, and total impulsiveness score (r=-0.272, p=0.035; r=-0.279, p=0.035, respectively). Right eye CMT and MMT values and BIS-11 attention-related impulsiveness scores were positively correlated (r=0.288, p=0.026; r=0.261, p=0.044, respectively) (Table 4).

**Table 4:** Clinical characteristics and OCT findings of patients with opiate use disorder

	Patient age	Undergoing treatment	Legal issue	BIS-11 motor	BIS-11 attention	BIS-11 making plans	BIS-11 total value
Macular layer							
Central macular thickness							
Right eye	0.267	0.196	0.012	0.131	0.288*	0.042	0.192
Left eye	0.257	0.153	0.313	0.046	0.204	0.036	0.121
Mean macular thickness							
Right eye	0.253	-0.074	-0.189	0.094	0.299*	0.088	0.213
Left eye	0.321	-0.083	-0.085	0.040	0.293*	0.003	0.160
Mean macular volume							
Right eye	-0.043	-0.039	0.089	-0.045	0.261*	0.026	0.111
Left eye	-0.012	-0.013	0.166	-0.072	0.215	-0.008	0.067
RNFL							
Superior quadrant							
Right eye	0.061	0.013	0.042	-0.165	-0.070	-0.046	-0.108
Left eye	-0.047	0.380*	-0.104	-0.055	-0.123	-0.171	-0.143
Inferior quadrant							
Right eye	0.004	-0.284	-0.151	-0.093	-0.124	-0.154	-0.160
Left eye	0.012	-0.153	-0.096	-0.193	-0.186	-0.114	-0.216
Temporal quadrant							
Right eye	0.035	0.026	0.062	-0.089	-0.101	-0.137	-0.131
Left eye	0.061	0.013	0.097	-0.085	-0.152	-0.177	-0.170
Nasal quadrant							
Right eye	0.016	0.035	-0.008	0.124	0.013	-0.190	0.040
Left eye	0.107	-0.179	0.004	0.150	-0.041	-0.165	0.035
Total value							
Right eye	-0.034	0.004	-0.143	-0.141	-0.272*	-0.141	-0.279*
Left eye	0.026	0.022	-0.035	-0.130	-0.201	-0.130	-0.202

BIS-11= Barratt Impulsiveness Scale; RNFL= retina nerve fiber layer. Pearson correlation analysis was used in the calculations. The value in the table is “r” values. *p<0.05.

## DISCUSSION

Our study was determined thin CMT, MMT, and MMV in patients with opiate use disorder compared with that of healthy controls. Moreover, it was also observed that a positive relationship was detected between the scores of macular thickness and volume and impulsiveness. In RNFL, thickening in the superior quadrant and total RNFL value was observed.

Our study is the first one to evaluate the OCT findings in patients with opiate use disorder. Upon review of the literature, the results obtained in limited OCT studies of patients with alcohol use disorder/SUD were contradictory^(11,13,14)^. In a study involving patients with alcohol use disorder, no differences were detected between the healthy controls and the patient group in terms of macular thickness and macular volume^(11)^. One study compared the OCT measurements of 17 patients with cocaine use disorder and 18 healthy controls. As a result, no differences were detected in the macular thickness and macular volume values of cocaine users’ healthy controls^13)^. Another study reported a case report of focal visual area defects that occurred due to macular neuroretinopathy after nasal cocaine intake^(14)^. Dopamine, which is the basic neurotransmitter in developing addiction, also has roles in vision signals and in ensuring the light adaptation in visual pathways^(15)^. It has been shown that the loss of dopamine is associated with the thinning of the macular layer^(16)^. The results of our study support these findings indirectly. It was determined that macular thickness and macular volumes in both eyes were thinned in patients with opiate use disorder compared to that in healthy controls. This was interpreted as the use of mutual neurotransmitter, the stimulation of dopamine receptors with opiate, and the inability to provide sufficient stimulation of visual pathways, thus, leading to thinning of the macula layer. Reduction of macular thickness and volume is characterized by reduced central visual acuity and progressive vision loss^17)^. Our study determined that participants had macular layer degeneration without impaired visual acuity and vision loss. Consequently, opiate users may be at risk of vision loss over time.

In the neurodevelopmental process, the retina and brain develop from a common biological origin, i.e., the ectoderm. Anatomically, the retina is an extension of the brain and part of the central nervous system, standing out as an important area for monitoring possible degenerations^((18)^. Further, dopamine neurotransmitters are involved in the basic modulation of the retina^((19)^. Previous studies revealed that dopamine is the main neurotransmitter involved in the development of opiate use disorder, and dopamine-2 receptor usability was lower than normal^((20)^. Based on this neurotransmitter similarity and the fact that the retina originates from a common embryological structure with the central nervous system, it is expected that changes are detected in OCT and RNFL values in psychiatric diseases. The results reported in the literature in those with psychiatric diseases regarding RNFL values are contradictory, as in the case in the macula layer^(11,12,13,14,21,22,23)^. Although publications in the literature report thinning^(21)^ in all RNFL quadrants in patients with schizophrenia, other studies indicate no observed differences with healthy controls^((22)^. In different studies conducted on patients with major depressive disorder and bipolar disorder, the RNFL thickness was not found to be different from that in healthy controls in all quadrants^(9,23)^. OCT findings were examined in patients with addiction in a limited number of studies^(11,12,13)^. It was reported in a previous study that thinning of the RNFL superior, inferior, and nasal quadrants was observed in cocaine addicts^(13)^. In another study examining OCT findings in patients with alcohol use disorder, although measured RNFL values were higher than that in healthy controls in superior quadrant, temporal quadrant, and total values, no statistically significant differences were detected^(11)^. Similarly, in our results, RNFL measurements were found to be thickened compared to that in healthy controls in certain quadrants. The RNFL superior quadrant and total RNFL values measured for both eyes were thinner in patients compared to that in healthy controls. When examined along with the common neurotransmitter hypothesis, it was shown that GABA, glutamate, and glycine, which play roles in addiction, were found in human visual pathways and retinal layers^(24)^. It was found in an animal study that glutamate levels changed in the retina after ischemia^(25)^. On the contrary, it was also shown that the change in the levels of these neurotransmitters in the central nervous system could cause disruption of stimuli in the retinal layers and damage to the retina^(26)^. In addition, opioid receptors were found in the retina^(27)^. In animal studies, it was also found that the stimulation of opioid receptors on the retina could change the retinal vascular tonus^(28)^.

Studies that evaluate psychiatric diseases and OCT findings report an association between clinical parameters such as disease duration and the number of hospitalizations and eye measurements^(9,10^). Studies conducted on patients diagnosed with obsessive-compulsive disorder and depressive disorder determined that changes in OCT measurements were associated with disease duration and the number of hospitalizations^(10)^. In our results, previous hospitalization was found to be associated with left eye RNFL superior quadrant thickness. No associations were detected between other OCT measurements and inpatient treatment, legal problems, and the patient’s age. In some studies in the literature, retinal thickness measured through OCT was found to be thinner with advancing age in the normal population^(29)^. In addition to the thinning with advancing age, studies were also conducted showing that retinal layers were thinner in women than that in men^(30)^. No associations were detected in our results between age and OCT findings. Furthermore, there were no intersex differences since all participants were males. Since the smoking status of our participants did not have statistically significant differences, no comparisons could be made in this respect.

In conclusion, it was determined that the CMT, MMT, and MMV values of patients diagnosed with opiate use disorder were reduced compared to that in healthy controls, and thickening in the RNFL superior quadrant and total RNFL values was observed. Based on the results obtained in this study, the clinical findings of patients with opiate use disorder may be accompanied by neurodegeneration findings or other visual-related disorders throughout their lives. Hence, when conducting clinical evaluations, follow-ups, and treatment plans for individuals with opiate use disorder, adding eye examinations at regular intervals will be beneficial.

Our study had several limitations. The relatively low number of samples, the cross-sectional nature of the study, and the fact that all participants were males are among the limitations. Although these limitations were adequate in making evaluations, they limited the generalizability and interpretability of our results. For our findings to gain importance, further longitudinal studies are needed in larger sampling groups, including neuroimaging techniques.
